# The journey of F1000Research since inception: through bibliometric analysis

**DOI:** 10.12688/f1000research.134244.1

**Published:** 2023-05-18

**Authors:** Dilip Kumar, Abhinav Kumar Shandilya, Sandeep Srivastava

**Affiliations:** 1 Welcomgroup Graduate School of Hotel Administration, Manipal Academy of Higher Education, Manipal, Karnataka, 576104, India; 2 Department of Hotel Management and Catering Technology, Birla Institute of Technology, Ranchi, Jharkhand, 835215, India; 3 Welcomgroup Graduate School of Hotel Administration, Manipal Academy of Higher Education, Manipal, Karnataka, 576104, India

**Keywords:** Bibliometric study, F1000Research, COVID-19, VOS-viewer, Biblioshiny, Bioinformatics, visualization.

## Abstract

**Background: **Bibliometric analysis is an approach adopted by researchers to understand the various analytics such as year-wise publications, their citations, most impactful authors and their contributions, identification of emerging keywords, multiple themes (niche, motor, basic, and emerging or declining) etc. F1000Research is one of the Q1 category journals that publishes articles in various domains, but a detailed journal analysis is yet to be done.

**Methods: **This study is an effort to extract the F1000Research journey information through bibliometric analysis using VOS-viewer and Biblioshiny (R-studio) interface. The F1000Research journal started its journey in 2012; since then, 5767 articles have been published until the end of 2022. Most of the published articles are from medical science, covering Biochemistry, Genetics & Molecular Biology, Immunology & Pharmacology, Toxicology & Pharmaceutics. To understand the research journey, various analyses such as publication & citation trends, leading authors, institutions, countries, most frequent keywords, bibliographic coupling between authors, countries and documents, emerging research themes, and trending keywords were performed.

**Results: **The United States is the biggest contributor, and COVID-19 is the most commonly occurred keyword.

**Conclusions: **The present study may help future researchers to understand the emerging medical science domain. It will also help the editors and journal to focus more on developing or emerging areas and to understand their importance towards society. Future researchers can contribute their quality research studies, focusing on emerging themes. These authors’ research can guide future researchers to develop their research area around the most impacted articles. They can collaborate with them to bring that emerging theme forward.

## Introduction

F1000Research is a journal under Taylor and Francis Group which has published 4947000+ articles and published by F1000 Research Ltd. It publishes a wide range of themes like social sciences, science, humanities, medicine, engineering and technology, agricultural and veterinary sciences, and arts without any biases by an editorial board. It publishes papers in multi-disciplinary areas and provides wider opportunities for researchers through its open-access publishing platform, offering rapid and regular publication. Compared to other journals ranked in the Q1 category, it is relatively new. However, in 11 years, it has published a vast quantity of research papers, i.e., 5767, until 31 ^st^ December 2022.

F1000Research publishes peer-reviewed research articles in a periodical order and has an International Standard Serial Number (ISSN) of 2046-1402. F1000Research started its publishing journey in 2012, having an H-index - 72, an impact factor (2021) - 3.23, an overall ranking of 4485, SCImago Journal Rank (SJR) – 0.939 ( Resurchify, 2023). SCImago Journal Ranking (SJI) has divided journals into four categories, namely Q1 (green) comprised of highest quality journals, Q2 is the second best quality journals, Q3 is the third best quality journal, and Q4 is the least quality journals ( SCImago, 2023).

F1000Research is a multi-disciplinary journal covering a vast publication domain; therefore, a bibliometric analysis was conducted to understand the journal’s past, present, and future research agenda to honour its stature. Bibliometric analysis help in measuring the journal’s progress journey through co-word, citation, and bibliographic coupling ( Donthu et al., 2020). Journal performance, development and content insights can be helpful for researchers in studying a specific journal ( Ratten et al., 2020). Recently, bibliometric analyses have been conducted in quality journals in the areas of nursing ( Yanbing et al., 2020), money laundering ( Saxena & Kumar, 2023), tourism ( Leong et al., 2021), hospitality ( Martorell Cunill et al., 2019), engineering ( Modak et al., 2020), medicare ( Lin et al., 2020), humanities ( Nwagwu & Egbon, 2011) and management ( Ratten et al., 2020). For every top-ranked journal, whether the “International Journal of Hospitality Management” or “International Marketing Review,” a bibliometric analysis study has been performed. However, a journal like F1000Research (a journal of great repute) has a bibliometric analysis not yet been conducted. This study aims to unveil the journey of F1000Research since its inception to understand its past, present, and future direction.

Various visualising software can be used for bibliometric analysis, such as VOS-viewer, Bibliometrix (R-studio), Gephi, CiteNet, Pajek, Sci ^2^, and HiteCite ( Van Eck & Waltman, 2013). In the present study, VOS-viewer and Biblioshiny (R-studio) are preferred over other visualising software due to their web-map pictorial representation, data-friendly features, and detailed analysis. This analysis helps in finding the research questions:

RQ1: What are the publication trends of F1000Research since its inception (2012-2022)?

RQ2: Who are the leading authors, organisations/institutions and countries publishing in the F1000Research journal?

RQ3: What are the most frequent keywords used and cluster formation based on keywords appeared?

RQ4: Which are the most cited publications and most impactful authors in the F1000Research journal?

RQ5: What is the major bibliographic coupling regarding countries, authors, documents, and organisations?

RQ6: What are the trending words and emerging research areas?

RQ7: What are the future research directions in the present research publication domain?

As F1000Research is a multi-disciplinary journal; it therefore covers a broad area of research publications that can help present, and future researchers and editors identify emerging areas, contribute their efforts to society, and widen the research horizon. This paper is organised into various parts such as the reasons behind choosing F1000Research journal for bibliometric analysis, methodology, results discussion generated from the VOS-viewer and Biblioshiny (R-studio) regarding the research questions (authors, country, publications, citations, bibliographic coupling, co-citations, and co-occurrence of author keywords), future directions, and implications, and limitations of study.

## Methods

### Study design

In 1969, Pritchard introduced Bibliometrics as “applying mathematical and statistical methods to books and other means of communication” ( Pritchard, 1969). In the present scenario, it is widely seen in every field. It is a quantitative and qualitative research analysis used to understand and highlight the impact of authors, institutions, collaborations, emerging research areas and countries. The present study used a bibliometric technique through VOS-viewer and Biblioshiny (R-studio) to analyse the performance of the F1000Research since its inception (2012). It is a Q1 category journal indexed in Scopus, UGC CARE, DOAJ, and PubMed. Scopus is one of the best reliable databases, which has 1.8+ billion cited references, 84+ million records, 17.6+ million authors’ profiles, 94.8+ affiliation profiles, and 7+ thousand publishers, where 35% of publications are from the field of social sciences, 27% physical sciences, 23% health sciences and 15% life sciences ( ELSEVIER, 2020). Hence, the Scopus database has been used to extract the metadata.

Metadata extracted from the Scopus database has been analysed using VOS-viewer software developed by van Eck & Waltman (2010) and Biblioshiny (R-studio) interface developed by Aria & Cuccurullo (2017). Bibliometric metadata represents various relationships, such as publications and citations ( Ding et al., 2017). The impact of the publications is measured through their citations, whereas the number of publications only quantifies their productivity ( Svensson, 2010). H-index explains “the number of papers with citation number >h, as a useful index to characterise a researcher’s scientific output” ( Hirsch, 2005), which has also been used in the present study.

### Data collection

Various studies have used keywords search index criteria ( Chen et al., 2017; Maier et al., 2020; Kumar et al., 2022; Leong et al., 2021; Pesta et al., 2018; Singh et al., 2022) for extracting the Scopus metadata. It has prepared the base and design for data collection, which has also been used in the present study.

Step 1: The Scopus database was searched with the keyword (ALL(F1000Research) AND PUBYEAR > 2011 AND PUBYEAR < 2023 AND (LIMIT-TO (EXACTSRCTITLE,“F1000research”)) AND (LIMIT-TO (LANGUAGE,“English”)) AND (LIMIT-TO (DOCTYPE,“ar”) OR LIMIT-TO (DOCTYPE,“re”))) in the ‘source title’ on 22 ^nd^ March 2023 from the IP address of ‘Manipal Academy of Higher Education, Manipal, India.’ Metadata of 72396 documents appeared in the initial search.

Step 2: Various journals appeared but were limited to an ‘F1000Research’ only., in which 5999 metadata appeared.

Step 3: The result from 2012-2022 (31 ^st^ December 2022), generated 5987 documents and then refined to the language ‘English,’ publication stage ‘Final’, limited to ‘article and review’ only generated 5767 documents.

Step 4: Only 5727 metadata were downloaded, whereas the remaining 40 could not be downloaded due to a metadata error. Finally, a CSV file, including “Citations information, bibliographical information, abstracts and keywords, funding details and other information,” was downloaded.

Step 5: Purification of data performed in CSV file using the conditional formatting features to remove the duplicate articles (202 articles) and removed seven articles due to retraction case. Finally, 5518 articles (metadata) were used for the final analysis using VOS-viewer software and Bibliometrix (R-studio) interface ( Kumar, 2023).

### Data analysis

The present study used VOS-viewer software version 1.6.19 and Biblioshiny (R-studio) 4.2.3 interface to analyse the F1000 research journey. A samples of 1000 papers considered as an good enough to generalise the result ( Rogers et al., 2020). To analyse the publication trends of F1000Research, find out the leading authors, organisations, countries, frequent keywords, most cited publications, bibliographic coupling between authors, organisations and countries, and emerging research areas since its inception VOS-viewer and Biblioshiny were used. VOS-viewer uses a fractional counting method, whereas the Scopus database generates metadata under a full counting system; therefore, full and fractional both counting methods have been used. However, Biblioshiny (R-studio) interface generates a detailed analysis. However, data reading showed poor keywords details, a completely missing number of cited references, and a completely missing science categories, which specifies some missing data. Although extracted metadata provided enough information to be used in the final analysis.

## Results

### Publication trends and citation analysis of F1000Research

**Figure 1.**
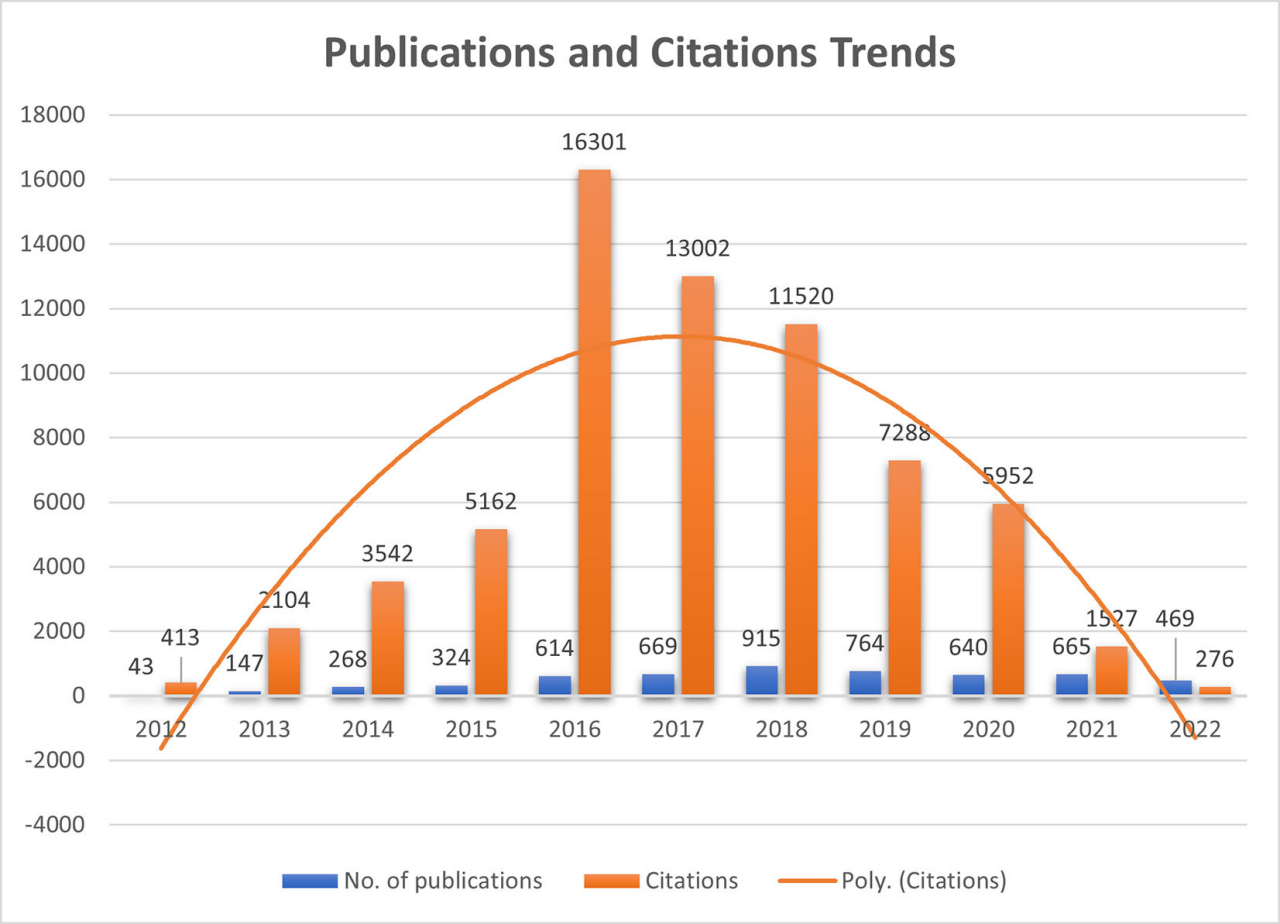
Publications and Citations trends since 2012 (Source: Scopus Database, Software: MS-excel).

Quantifying publications and their citations play a significant role in assessing the journal’s global presence and recognition. In the present study, Figure 1 represents the number of publications that remained almost constant during 2016, 2017, 2020, and 2021 (ranging between 614-669), whereas, in 2018, publications increased drastically to 915 and again reduced in 2019 to 764. It shows that before COVID-19, upward growth in the publication was seen due to the journal’s recognition and popularity among the researchers. Citations which make the article journal more impactful, increased dramatically in 2016 to 16301 from 5162 (2015) and again started declining. Citations formed a bell-curve shape, which shows that they grew from 413 citations in 2012 to 16301 and reduced to 276 in 2022. New articles are still to create their impact in the researcher’s mind to cite them in their literature. F1000Research started its journey in 2012 and published only 43 articles, multiplied thrice in consecutive years.

**Table 1.** Procedure of data collection (step by step).

Steps used	The command used to extract the data	Data extracted
Step 1	F1000Research	72,396
Step 2	Limit to – F1000Research only	5999
Step 3	Limit to – Year (2012-2022), English, Final Publication stage, articles & reviews	5767
Step 4	An error occurred while downloading the metadata; we downloaded 40 less	5727
Step 5	Data purification – Removal of duplicate and retraction data	5518

### Organisations contributions (Documents & Citations)

**Figure 2.**
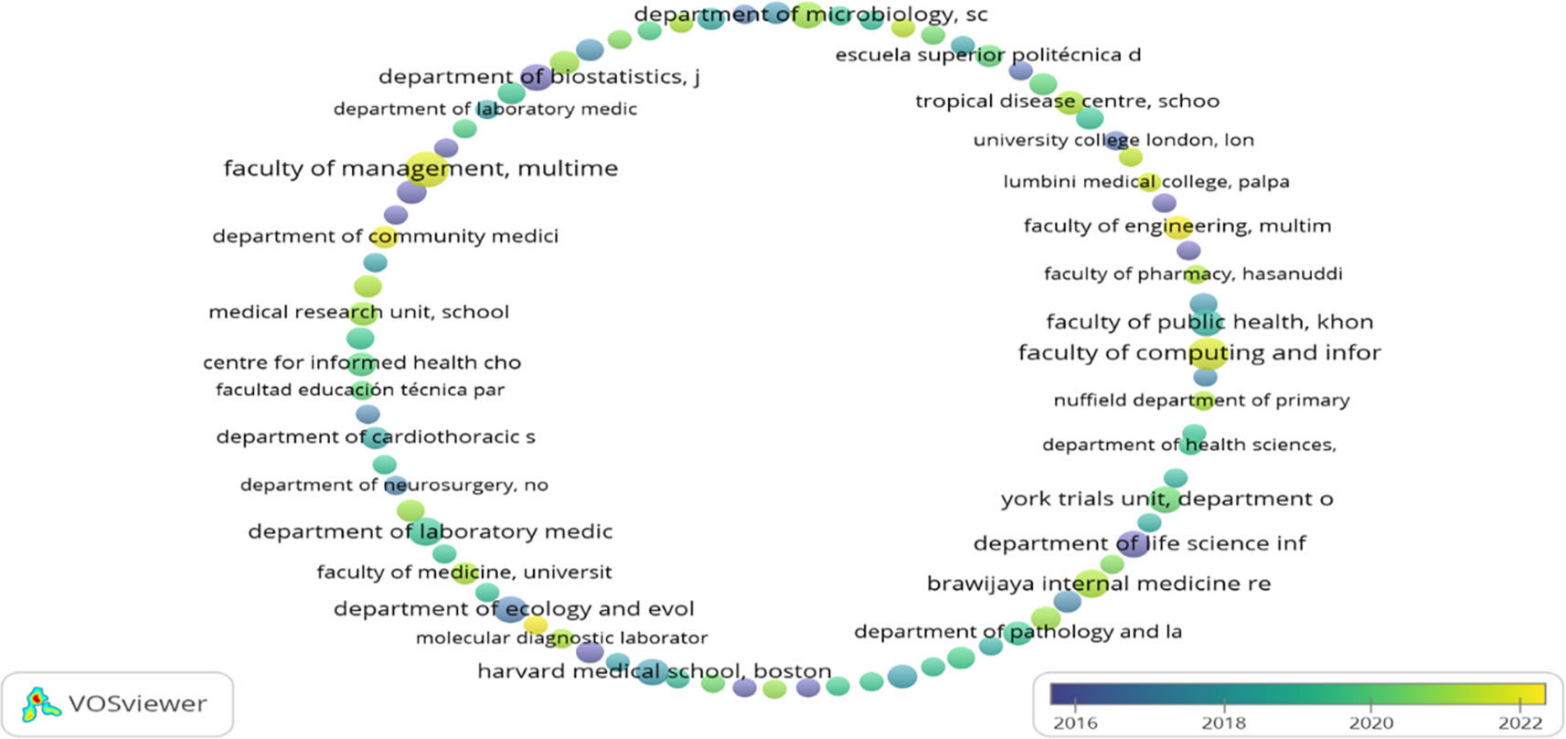
Overlay graphical representations of the top organisation (Data source: Scopus, Software: VOS-viewer).

Table 2 represents the top 20 organisations that contributed to the F1000Research journal. A maximum number of articles published by “Faculty of Management, Multimedia University, Cyberjaya, Selangor, Malaysia,” i.e., 12 but got only seven citations as they have published their work recently (2021). Whereas the maximum number of citations received by “SIB Swiss Institute of Bioinformatics, University of Zurich, Zurich, Switzerland,” i.e., 1834, is exhibited in Figure 2. However, it has published only four articles. Following the “Department of Microbiology, School of Medicine, Universitas Syiah Kuala, Banda Aceh, Aceh, Indonesia” published seven articles having a citation of 211. “Brawijaya Internal Medicine Research Center, Department of Internal Medicine, Faculty of Medicine, Universitas Brawijaya, Malang, East Java, Indonesia”, ranked number three, has published eight documents but has g-citations of 202.

**Table 2.** Top 20 organisations contributing to F1000Research (Source: Scopus database, Software: VOS-viewer & MS-excel).

Organisation	Documents	Citations	Citations/documents
“SIB Swiss Institute of Bioinformatics, University of Zurich, Zurich, 8057, Switzerland”	4	1834	459
“Department of Microbiology, School of Medicine, Universitas Syiah Kuala, Banda Aceh, Aceh, 23111, Indonesia”	7	211	30
“Brawijaya Internal Medicine Research Center, Department of Internal Medicine, Faculty of Medicine, Universitas Brawijaya, Malang, East Java, 65145, Indonesia”	8	202	25
“Medical Research Unit, School of Medicine, Universitas Syiah Kuala, Banda Aceh, Aceh, 23111, Indonesia”	6	199	33
“Harvard Medical School, Boston, MA, United States”	7	183	26
“Department of Biostatistics, Johns Hopkins Bloomberg School of Public Health, Baltimore, 21205, MD, United States”	7	134	19
“Tropical Disease Centre, School of Medicine, Universitas Syiah Kuala, Banda Aceh, Aceh, 23111, Indonesia”	6	121	20
“Department of Pharmacy, BGC Trust University Bangladesh, Chittagong, 4381, Bangladesh”	5	116	23
“Collaborative Drug Discovery, Burlingame, 94010, CA, United States”	4	114	29
“Department of Life Science Informatics, B-IT, Limes Program Unit Chemical Biology and Medicinal Chemistry, Rheinische Friedrich-Wilhelms-Universität, Bonn, D-533, Germany”	7	109	16
“Faculty of Medicine, Universitas Brawijaya, Malang, East Java, 65117, Indonesia”	5	109	22
“Faculty of Pharmacy, Hasanuddin University, Makassar, South Sulawesi, 90245, Indonesia”	4	108	27
“University College London, London, Wc1e 6bt, United Kingdom”	4	95	24
“Molecular Diagnostic Laboratory, Johns Hopkins Aramco Healthcare, Dhahran, 31311, Saudi Arabia”	4	91	23
“Department of Biomedical Dental Sciences, College of Dentistry, Imam Abdulrahman Bin Faisal University, Dammam, 31441, Saudi Arabia”	4	90	23
“Earlham Institute, Norwich Research Park, Norwich, United Kingdom”	4	87	22
“The Donnelly Centre, University of Toronto, Toronto, M5s 3e1, ON, Canada”	5	70	14
“Gladstone Institutes, San Francisco, 94158, CA, United States”	4	69	17
“The Genome Analysis Centre, Norwich Research Park, Norwich, Nr4 7uh, United Kingdom.”	5	58	12
“Department of Medicine, University of California, San Diego, 92093-0688, CA, United States”	5	57	11

European, Asian (mainly Malaysia, Indonesia, and Saudi Arabia), and American (both south and north) organisations contributed to the top 20 organisations list. In contrast, African and Australian organisations are not in the top 20 organisations listed in Table 2. The organisation that published their articles in the F1000Research journal from 2016-2022 (22 ^nd^ March) is represented in Figure 2. Organisations marked in yellow (“Faculty of Computing and Informatics,” “Faculty of Management,” “Faculty of Engineering,” “Molecular Diagnostic Laboratory,” and “Department of Community Medicine”) are relatively young in publishing their work in the F1000Research journal.

### Country’s production overtime

**Figure 3.**
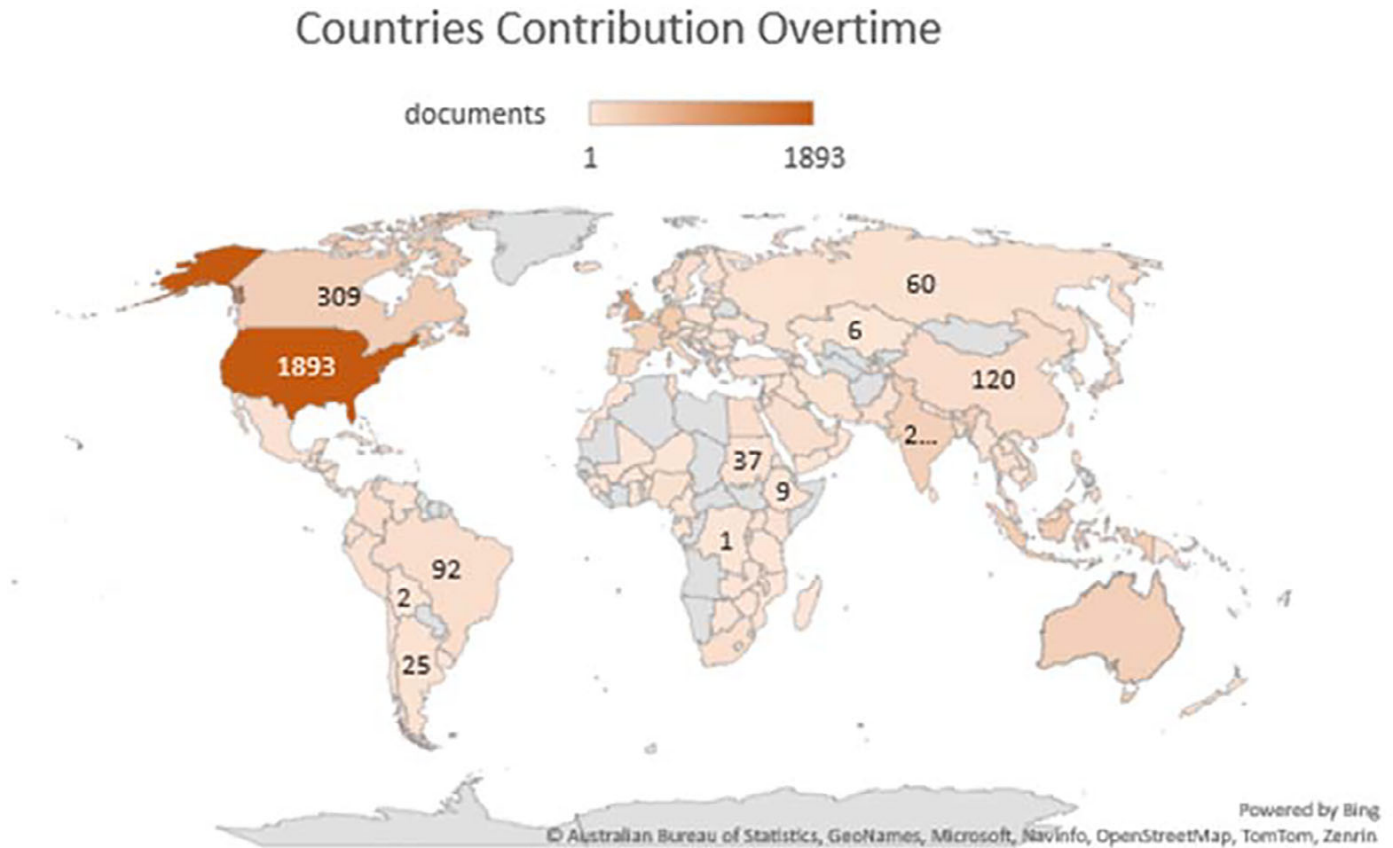
Graphical representation of countries’ contribution to the F1000Research journal (Data source: Scopus, Software: MS-excel).

Table 3 represents the country’s production over time, citations generated by the specific country and total link strength (TLS). Figure 3 illustrates the significant countries’ contributions/production over time (2012-2022). According to Worldometer (2023), there are 195 countries in the world, of which 164 countries have contributed their work to the F1000Research journal, ranging from 1 to 1893 publications. A major contribution was from the United States, United Kingdom, and Germany, respectively, i.e., 1893, 882 and 357. However, citations from the United States, United Kingdom, and Canada ranked in the top three (31765, 12866 and 5776, respectively) as shown in Table 3. India has also contributed 274 publications (7 ^th^ in publication ranking), citations of 1589 (13 ^th^ in citations ranking) and a TLS of 17025. A major chunk of Africa, Central Asia, and Greenland have contributed their work to the F1000Research journal, as shown in Figure 3. For global recognition and contribution, the journal should target the countries whose contribution is either less or not contributed. Quality publications can come from even the world’s smallest countries; therefore, more focus must be given to those countries.

**Table 3.** Country’s overtime production (Data source: Scopus, Software: VOS-viewer & MS-excel).

Country	Documents	Citations	Total link strength
United States	1893	31765	99249
United Kingdom	882	12866	73643
Canada	309	5776	33610
Germany	357	5670	48765
Australia	281	5129	31965
Switzerland	192	4428	34953
Italy	208	3145	24442
France	242	2728	35556
Netherlands	152	2182	32360
China	120	2130	21390
Belgium	102	1937	24602
Spain	163	1641	33496
India	274	1589	16969
Sweden	95	1271	21112
Indonesia	300	1261	12077
Denmark	74	1172	16440
Brazil	92	1096	8477
Mexico	51	966	5612
Japan	127	960	12699
Bangladesh	58	855	5645

### Most frequent keywords and their occurrence

**Figure 4.**
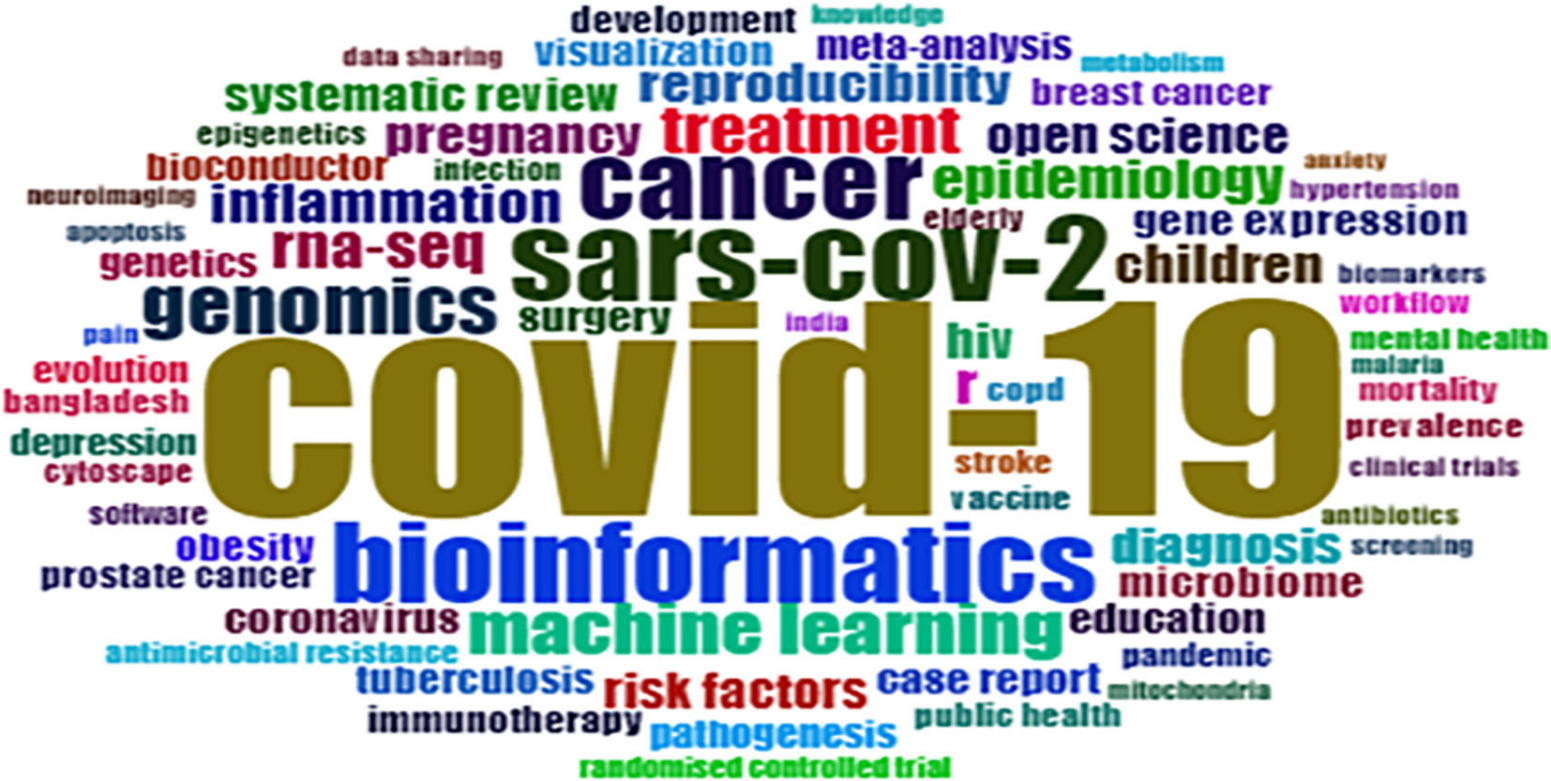
WordCloud of most frequent keywords (Software: Biblioshiny (R-studio)).

The most frequent keyword visualisation shown in Figure 4 has been generated using the Biblioshiny (R-studio) interface. These keywords are based on the co-occurrence of author keywords, which helps understand the most impactful keywords and keyword popularity. Table 4 exhibits the top 20 most occurred, strongly linked, and high-impact keywords. Future researchers use highly cited keywords as they receive global attention very fast as compared to least cited keywords. Keywords such as “COVID-19,” “Bioinformatics,” and “SARS-Cov-2” should be used along with “Proteinaceous,” “Peru,” “Screening,” “Oxidative stress,” etc., to explore the new facts and relationships. Countries like India, Nepal, and Bangladesh (Asian countries) have linkages with the “COVID-19” keyword. Bibliometric analysis can be an essential tool to detect research trends for the present and future ( Pesta et al., 2018).

**Table 4.** Top-20 author keywords co-occurrences (Data source: Scopus, Software: VOS-viewer & MS-excel).

Rank	Keyword	Occurrences	Total link strength
1	COVID-19	183	165
2	SARS-CoV-2	76	95
3	Bioinformatics	69	65
4	Cancer	66	36
5	Genomics	48	55
6	Machine Learning	44	33
7	Treatment	40	26
8	RNA-S	37	33
9	Inflammation	35	25
10	R	35	35
11	Children	33	22
12	Epidemiology	33	31
13	Reproducibility	33	31
14	Pregnancy	32	20
15	Diagnosis	31	21
16	HIV	31	11
17	Risk Factors	31	24
18	Open Science	29	27
19	Systematic Review	29	25
20	Gene Expression	27	23

### Author keywords co-occurrence analysis

The fine-grained tropical structure is better understood by combining the keywords and cited references in the research field which also helps develop the relationships between various topics and their sub-topics ( Van den Besselaar & Heimeriks, 2006). VOS-viewer software helps identify the themes using the keywords co-occurrence analysis in the specific study area ( van Eck & Waltman, 2020), resulting in bibliographic clusters and emerging and least explored themes ( Donthu et al., 2020).

**Figure 5.**
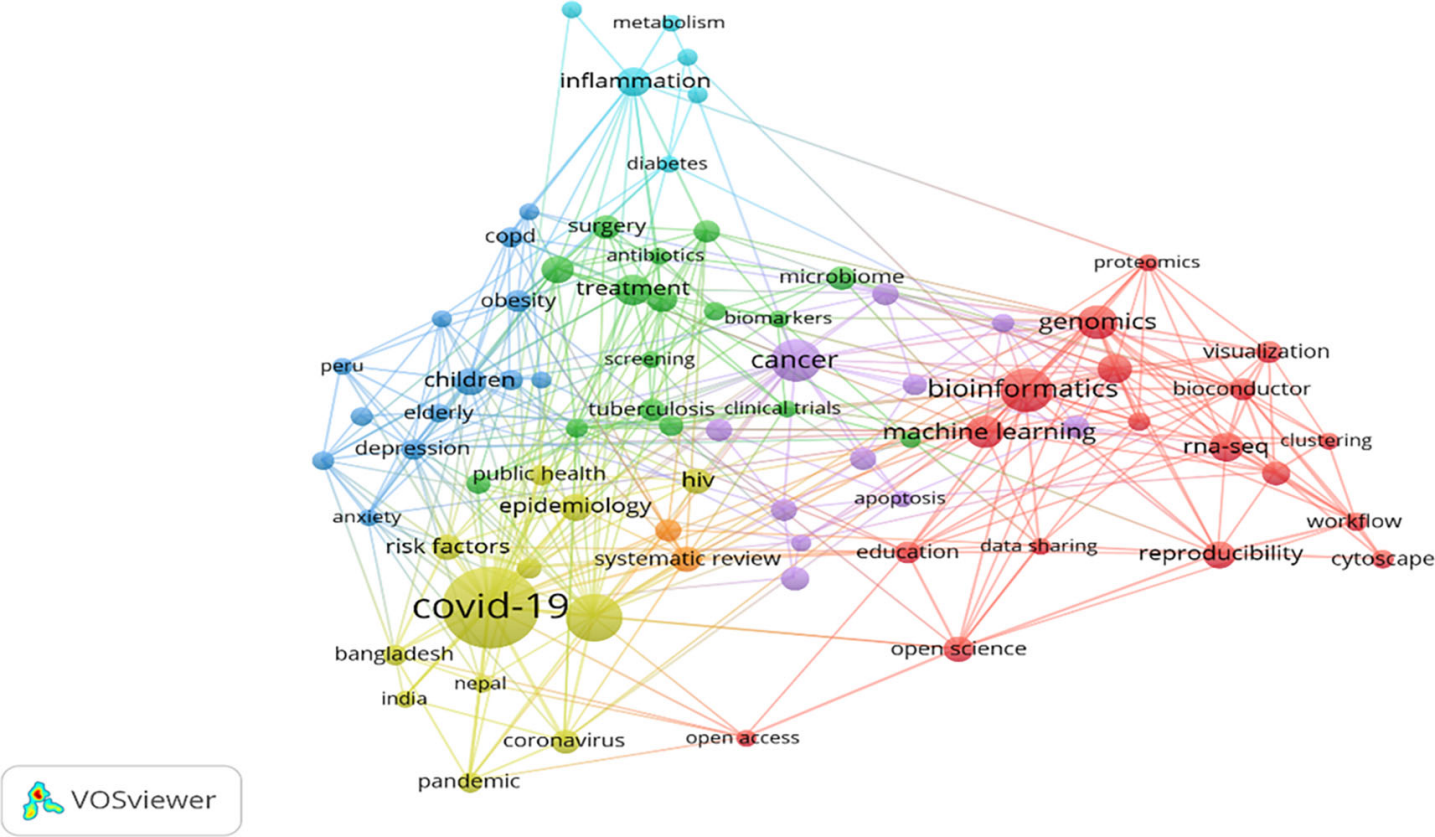
Author keywords co-occurrence and TLS visualisation (Data source: Scopus, Software: VOS-viewer).

1414 author keywords were found in the metadata extracted from the Scopus database. Only 78 keywords met the desired threshold when the minimum number of co-occurrences was restricted to 15 keywords. Figure 5 shows the visualisation of highly appeared keywords in various clusters marked in red (18 keywords), green (16 keywords), blue (13 keywords), yellow (12 keywords), purple (11 keywords), cyan (6 keywords), and orange (2 keywords) colours in the F1000Research journal since its inception. The red cluster is the most dominating and impactful cluster, whereas the orange is the least impactful cluster. Table 5 exhibits the 78 keywords that appeared in various clusters (depicted seven themes). These clusters emerged as different themes such as “bioinformatics” (69 occurrences), “treatment” (40 occurrences), “children” (33 occurrences), “COVID-19” (183 occurrences), “cancer” (66 occurrences), “inflammation” (35 occurrences) and “systematic review” (29 occurrences).

**Table 5.** Keywords co-occurrences cluster formation (Data source: Scopus, Software: VOS-viewer & MS-excel).

Keywords	Occurrences	Total link strength
*Cluster 1*		
Bioinformatics	69	65
Genomics	48	55
Machine Learning	44	33
RNA-Seq	37	33
R	35	35
Reproducibility	33	31
Open Science	29	27
Gene Expression	27	23
Visualisation	25	25
Bioconductor	24	45
Education	24	22
Cytoscape	19	13
Workflow	19	28
Software	18	14
Data Sharing	17	14
Clustering	15	12
Open Access	15	13
Proteomics	15	18
*Cluster 2*		
Treatment	40	26
Pregnancy	32	20
Diagnosis	31	21
Microbiome	27	9
Surgery	26	10
Tuberculosis	25	11
Pathogenesis	24	16
Mortality	20	16
Vaccine	20	12
Antimicrobial Resistance	19	9
Infection	19	21
Antibiotics	17	11
Biomarkers	17	10
Clinical Trials	17	12
Neuroimaging	16	12
Screening	15	6
*Cluster 3*		
Children	33	22
Obesity	25	16
Depression	22	35
COPD	21	14
Stroke	20	11
Elderly	19	10
Mental Health	19	22
Randomised Controlled Trial	18	4
Hypertension	17	11
Knowledge	16	6
Anxiety	15	23
Asthma	15	15
Peru	15	15
*Cluster 4*		
COVID-19	183	165
SARS-CoV-2	76	95
Epidemiology	33	31
HIV	31	11
Risk Factors	31	24
Coronavirus	26	38
Bangladesh	21	19
Prevalence	21	25
Public Health	21	13
Pandemic	20	29
India	17	11
Nepal	15	9
*Cluster 5*		
Cancer	66	36
Case Report	26	5
Genetics	24	17
Breast Cancer	23	8
Development	23	6
Immunotherapy	23	7
Prostate Cancer	23	10
Evolution	22	7
Epigenetics	19	11
Apoptosis	16	11
Malaria	16	10
*Cluster 6*		
Inflammation	35	25
Pain	17	6
Metabolism	16	3
Mitochondria	16	5
Diabetes	15	7
Oxidative Stress	15	3
*Cluster 7*		
Systematic Review	29	25
Meta-Analysis	24	20

In Figure 5, keywords are depicted by a node whose size specifies occurrences ( van Eck & Waltman, 2020). Bigger the node size, the greater the occurrences of the keyword. Total link strength (TLS) has been represented by the line thickness between the nodes, representing the keyword’s co-occurrence frequency within the links exhibited in Table 5.

*Cluster 1 (Red) = Bioinformatics.* This cluster comprises 18 keywords and is the most significant among the 7 clusters. “Bioinformatics” was found as the maximum occurred keyword having 69 appearances and the maximum TLS of 65 exhibited in Table 5. It is well connected with “genomic,” “machine learning,” “RNA-Seq,” “R,” “Reproducibility,” “open science”, and so on. It shows that the F1000Research journal mainly covers medical science articles where the “bioinformatics” keyword plays a significant role. “Bioinformatics” is over 50 years old and recently emerged to support next-gen data analysis. It has faced multiple challenges recently while managing big data and the reproducibility of results, which can help integrate the same into academics ( Gauthier et al., 2019). To extract information from big data, various machine-learning algorithms are widely used and applied in bioinformatics ( Min et al., 2017). Machine learning has revolutionised computational biology (part of bioinformatics) in transforming massive data through technology into knowledge which can help understand genomic, proteomics and system biology ( Larrañaga et al., 2006). This cluster discusses the science involved in handling bioinformatics and their application in improving education through software and increasing visibility through open science.

*Cluster 2 (Green) = Treatment.* This cluster comprises 16 keywords represented in Table 5 has shown “treatment” as a maximum occurred keyword (40 appearances) and TLS (40). It is well connected with various keywords such as “pregnancy,” “diagnosis,” “surgery,” “microbiome,” “tuberculosis,” “vaccine”, and so on. This cluster discusses the treatment of various diseases, infections, vaccines, and clinical trials. Pregnant women will continue to be a priority group for treatment optimisation in the era of compulsory treatment as treatment duration and antiretroviral therapy (ART) alternatives increase, promoting both their health and the health of their kids exposed to ART ( Bailey et al., 2018). Diagnosis and treatment go hand in hand; therefore, these two words are in the same cluster.

Cluster 3 (blue) *= Children.* This cluster comprises 13 keywords represented in Table 5. The maximum occurred keyword is “children”, having 33 occurrences and a TLS of 22. TLS denotes that “children” is linked up with 22 different keywords. Significant linkages are “obesity,” “depression,” “COPD,” “stroke,” “elderly,” “mental health”, and so on. This cluster discussed the disease and its impact on mental health and well-being. Obesity has become a global health problem, becoming common due to increased screen time ( World Health Organization, 2005). The advertisement of junk and fast food impacts children’s minds towards that food and ultimately results in obesity ( Strasburger et al., 2011). Therefore, this cluster needs special attention.

*Cluster 4 = COVID-19.* This cluster has 12 keywords, and these are highly correlated to COVID-19. COVID-19 has the maximum number of occurrences, i.e., 183 and TLS of 165 in the F1000Research journal. None of the keywords have even 100 occurrences, whereas COVID-19 has 183, which shows the impact of COVID-19-related publications in the journal. “COVID-19” has been linked with “SARS-CoV-2,” “epidemiology,” “HIV” “risk factors,” “coronavirus,” “Prevalence,” “public health” “pandemic,” “Bangladesh,” “Nepal,” and “India” exhibited in Table 5. SARS-CoV-2 spread globally and was declared a pandemic by World Health Organisation. It damaged health and wealth and increased poverty globally. A review proposed by Ciotti et al. (2020) provided information regarding epidemiology, its origin, infection to humans and safety issues. Various researchers ( Pokhrel & Chhetri, 2021) have also contributed to digitally improving the teaching-learning process to fight against a COVID-19-like pandemic in future. This cluster mainly focuses on the COVID-19 pandemic and its impact on developing countries like India, Bangladesh, and Nepal.

*Cluster 5 = Cancer.* The fifth cluster consists of 11 keywords, clustered around the “Cancer” keyword, 66 occurrences and 36 TLS exhibited in Table 5. “Case report,” “genetics,” “breast cancer,” “development,” and “immunotherapy” are the significant keywords strongly linked with the “cancer” keyword. This cluster mainly focuses on a widely spreading disease, “Cancer”, which must be prevented early rather than curing the advanced stage ( Nixon et al., 1973). Due to the increase of cancer worldwide, cancer immunotherapy's impact has gained popularity in cancer clinical care ( Blank et al., 2016). To combat this deadly disease, Silverstein et al. (2006) insisted on understanding the relationship between cancer and the immune system to develop treatment options for patients. Similar genomic profiles of tumors can help apply therapies to cancer types ( Hegde & Chen, 2020).

*Cluster 6 = Inflammation.* This cluster comprises six keywords. The most impactful keyword in this cluster is “inflammation”, have 35 occurrences and a TLS of 25 exhibited in Table 5. The remaining five keywords, “pain,” “metabolism,” “mitochondria,” “diabetes,” and “oxidative stress,” are highly linked with the most impactful keyword, “inflammation.” This cluster tried to explain the symptoms and the diseases. Many chronic inflammatory diseases are caused due to imbalance of mitochondria, metabolism and inflammation ( Tschopp, 2011). Inflammation can be a possible mechanism and prevent diabetes in type 1 and type 2 ( Tsalamandris et al., 2019). Inflammation and oxidative stress are affected by hypertension and increase blood pressure regardless of medicine use ( Pouvreau et al., 2018).

*Cluster 7 = Systematic review.* This is the smallest cluster that has only two keywords. The “Systematic review” keyword has 29 occurrences and 25 TLS, whereas “meta-analysis” has 24 occurrences and 20 TLS exhibited in Table 5. These two keywords are used as powerful tools for overcoming the handling of large-scale data. They can help present results from different studies conducted on a similar topic. A thorough understanding of meta-analysis is required to understand and accept the conclusions of various studies ( Ahn & Kang, 2018).

### Overlay visualisation of author keywords

**Figure 6.**
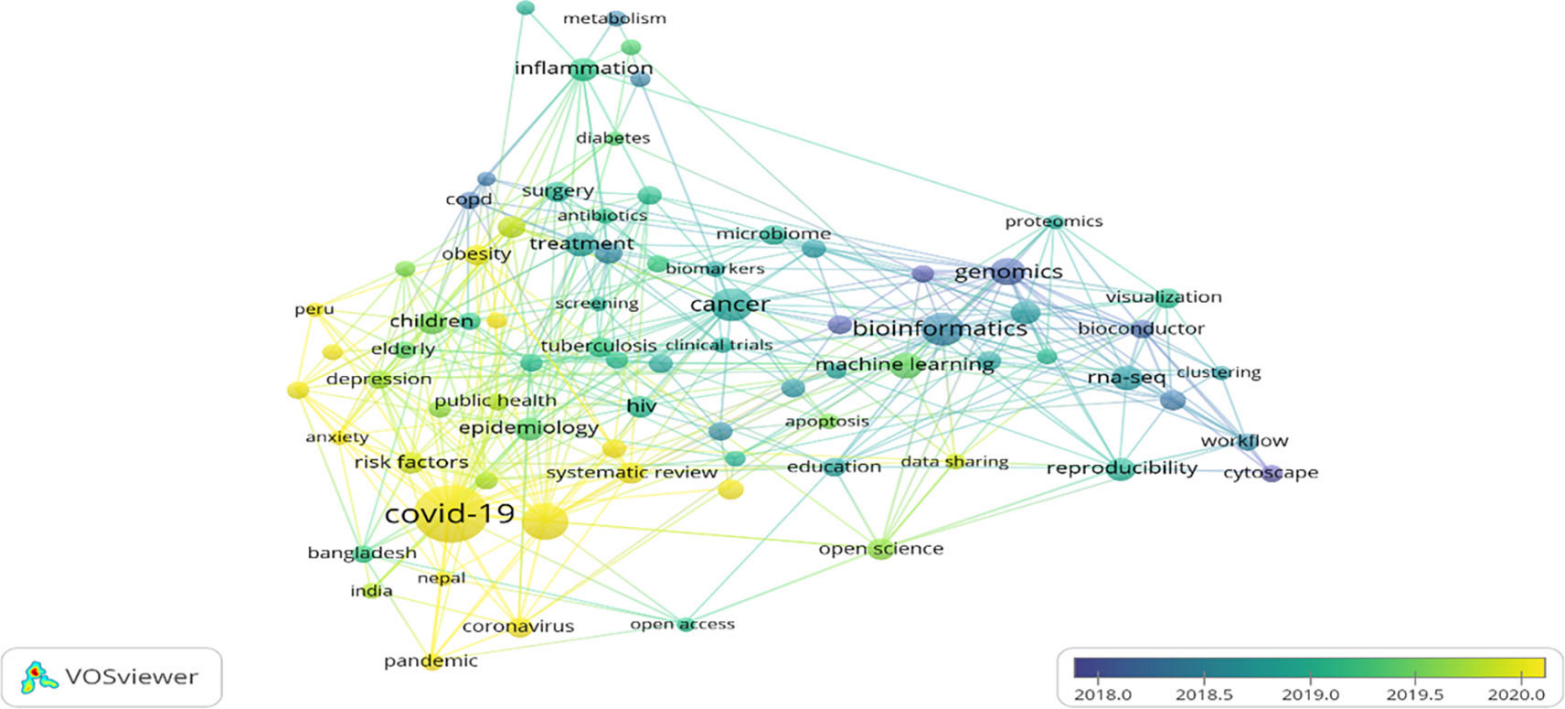
Overlay visualisation of author keywords (Data source: Scopus, Software: VOS-viewer).

Overlay visualisation of author keywords co-occurrences highlights the old and latest keywords through a bibliometric web map ( van Eck & Waltman, 2010). Figure 6 exhibits the five clusters marked in five colours ranging from purple to yellow. Purple colours depicted the old keywords, such as “Genomics” “and Cytoscape,” whereas the latest keywords appeared yellow colour such as “COVID-19,” “Anxiety,” “Coronavirus,” “Obesity,” “Risk Factors,” and “Depression” are latest keywords. The latest and most popular keyword in 2020 was “COVID-19”; in the middle of 2019, the most popular keyword was “Machine learning.” It shows that COVID-19 fever is not yet over; researchers are still exploring this area as much as possible to fight against the deadly pandemic and to bring life back to normal.

### Documents-citation analysis

The citation analysis explains the highly cited documents and authors ( Waltman et al., 2020) in the specific journal, performed through VOS-viewer software. Analysis was performed by restricting a document's minimum number of citations to 100. Out of 5518 documents, only 71 met the desired threshold. Table 6 exhibited the top 20 authors and articles with maximum citations in F1000 research journals. 71 documents with more than 100 citations which the previous researchers have used and now can be used in theoretical concepts for future studies. “Differential analyses for RNA-seq: transcript-level estimates improve gene-level inferences”, authored by Soneson et al. (2015), has a maximum citation of 1570, which is almost thrice more than the second highest cited document (574 citations), i.e., “FastQ Screen: A tool for multi-genome mapping and quality control” authored by Wingett & Andrews (2018). “Leishmaniasis: A review”, authored by Torres-Guerrero et al. (2017), has 516 citations, “Bioconductor workflow for microbiome data analysis: from raw reads to community analyses” authored by Callahan et al. (2016) has 439 citations and “Current understanding of Alzheimer’s disease diagnosis and treatment” authored by Weller & Budson (2018) has 434 citations. These documents can prepare a base for future researchers to develop a robust theory.

**Table 6.** Top 20 highly cited documents (Data source: Scopus, Software: VOS-viewer & MS-excel).

Rank	Document	Author/s	Citations
1	“Differential analyses for RNA-seq: transcript-level estimates improve gene-level inferences”	( Soneson et al., 2015)	1570
2	“FastQ Screen: A tool for multi-genome mapping and quality control”	( Wingett & Andrews, 2018)	574
3	“Leishmaniasis: A review”	( Torres-Guerrero et al., 2017)	516
4	“Bioconductor workflow for microbiome data analysis: from raw reads to community analyses”	( Callahan et al., 2016)	439
5	“Current understanding of Alzheimer’s disease diagnosis and treatment”	( Weller & Budson, 2018)	434
6	“A step-by-step workflow for low-level analysis of single-cell RNA-seq data with Bioconductor”	( Lun et al., 2016)	396
7	“Epidemiology of mental health problems in COVID-19: A review”	( Hossain et al., 2020)	379
8	“Plant adaptation to drought stress”	( Basu et al., 2016)	376
9	“From reads to genes to pathways: differential expression analysis of RNA-Seq experiments using Rsubread and the edgeR quasi-likelihood pipeline”	( Chen et al., 2016)	356
10	“Thousands of exon skipping events differentiate among splicing patterns in sixteen human tissues”	( Florea et al., 2013)	313
11	“Prediction of the SARS-CoV-2 (2019-nCoV) 3C-like protease (3CL pro) structure: virtual screening reveals velpatasvir, ledipasvir, and other drug repurposing candidates”	( Chen et al., 2020)	312
12	“taxize: taxonomic search and retrieval in R”	( Chamberlain & Szöcs, 2013)	284
13	“Recent advances in (therapeutic protein) drug development”	( Lagassé et al., 2017)	279
14	“HiCUP: pipeline for mapping and processing Hi-C data”	( Wingett et al., 2015)	273
15	“The academic, economic and societal impacts of Open Access: an evidence-based review”	( Tennant et al., 2016)	261
16	“A step-by-step workflow for low-level analysis of single-cell RNA-seq data with Bioconductor”	( Lun et al., 2016)	251
17	“Comprehensive comparison of Pacific Biosciences and Oxford Nanopore Technologies and their applications to transcriptome analysis”	( Weirather et al., 2017)	240
18	“RNA-seq analysis is easy as 1-2-3 with limma, Glimma and edgeR”	( Law et al., 2016)	234
19	“CoNet app: inference of biological association networks using Cytoscape”	( Faust & Raes, 2016)	226
20	“Dietary assessment methods in epidemiological research: current state of the art and future prospects”	( Naska et al., 2017)	213

### Most impactful authors

During the review of the F1000Research journal, 5290 authors were identified. While checking the citations analysis through VOS-viewer and limiting the maximum number of authors per document to 25 and the minimum number of documents of an author to two, only 225 authors met the desired threshold. Table 7 exhibits the top 20 authors, affiliations, country, total publications, total citations, and average cited documents since the journal’s evolution until 2022. Quantifying in publications is one of the criteria for increasing visibility on a global platform. However, it is challenging to gain popularity without citations of specific documents. Mainly European, Asian, and American continent authors’ visibility was found in the top-20 authors in terms of quantification of publication, whereas African and Australian authors did not appear in this list. The leading author’s impact is exhibited in Table 8 based on the h-index, g-index, and m-index. Various algorithms have been developed to calculate the most impactful author based on the publications, citations, author’s visibility, and continuity of publications. The g-indexed were designed to measure the global citation performance of articles or authors. It is also known as an improved version of the h-index ( Egghe, 2006). The m-index is a metric used to measure the h-index divided by the start of the publication year until the latest year.

**Table 7.** Author, affiliation, countries, publications, and citation analysis (Data source: Scopus, Software: VOS-viewer & MS-excel).

Name of author	Organisations	Country	NP	TC	ACD
Jürgen Bajorath	“Life Science Informatics, University of Bonn”	Germany	19	222	12
Jonny Karunia Fajar	“ Brawijaya Internal Medicine Research Center, Universitas Brawijaya”	Indonesia	17	277	16
Ben Busby	“National Center for Biotechnology Information, National Library of Medicine, National Institutes of Health, 8600 Rockville Pike, Bethesda, MD”	USA	17	65	4
Jan G. Jakobsson	“Department of Anaesthesia & Intensive Care, Institution for Clinical Sciences, Danderyds University Hospital, Karolinska Institutet, Stockholm, 18288”	Sweden	14	119	9
Harapan Harapan	“Medical Research Unit, Tropical Diseases Center, Department of Microbiology, Universitas Syiah Kuala”	Indonesia	13	273	21
Muhammad Ilmawan	“Faculty of Medicine, Universitas Brawijaya, Malang, East Java, 65145”	Indonesia	13	246	19
Gary D. Bader	“Molecular Genetics and Computer Science, The Donnelly Centre, University of Toronto”	Canada	12	577	48
Sean Ekins	“Collaborations Pharmaceuticals, Inc. (356). MEB, 1 RWJ Place CN19”	USA	12	269	22
Joseph F. John, Jr.	“Robert Wood Johnson Medical School, New Brunswick, New Jersey”	USA	11	111	10
Alexander R Pico	“Institute of Data Science and Biotechnology, Gladstone Institutes, San Francisco, CA”	USA	11	177	16
Arunima Biswas	“Department of Physiotherapy, Manipal College of Health Professions, Manipal Academy of Higher Education, Manipal, Karnataka”	India	10	89	9
Firzan Nainu	“Faculty of Pharmacy, Hasanuddin University, Tamalanrea, Makassar, South Sulawesi, 90245”	Indonesia	10	237	24
Yee Wan Lee	“Faculty of Management, Multimedia University, Cyberjaya, Selangor, 63100”	Malaysia	9	102	11
David Moher	“School of Epidemiology and Public Health, University of Ottawa, & Centre for Journalology, Ottawa Hospital Research Institute Ottawa, Ontario”	Canada	9	180	20
Yiwei Wang	“Center for Innovation in Brain Science, University of Arizona, Tucson, AZ, 85721”	USA	9	366	41
Helnida Anggun Maliga	“Faculty of Medicine, Universitas Brawijaya, Malang, East Java”	Indonesia	8	125	16
Ali A. Rabaan	“Molecular Diagnostic Laboratory, Johns Hopkins Aramco Healthcare, Dhahran”	Saudi Arabia	7	208	30
Alfonso J. Rodriguez-Morales	“Public Health and Infection Research Group, Faculty of Health Sciences, Universidad Tecnológica de Pereira, Pereira, Risaralda”	Colombia	7	172	25
Sweta Singh	“Savitribai Phule Pune University, Pune, India”	India	7	90	13
Aaron Lun	“Genentech, Inc: South San Francisco, CA”	US	6	1052	175

**Table 8.** Most impactful authors in F1000Research journal (Data source: Scopus, Software: VOS-viewer & MS-excel) h-index = metric used to quantify the scholarly output; g-index = a measure of researcher-specific impact; m-index = The h-index divided by the active number of years by the author; TC = Total citations; TN = Total number of publications; PY-start = Publication starting the year.

Rank	Name of author	h-index	g-index	m-index	TC	NP	PY-start
1	Gary D. Bader	9	12	0.9	577	12	2014
2	Sean Ekins	9	12	0.9	269	12	2014
3	Jürgen Bajorath	8	14	0.667	222	19	2012
4	Jonny Karunia Fajar	8	16	1.6	277	17	2019
5	Harapan Harapan	8	13	1.6	273	13	2019
6	Arunima Biswas	7	9	0.875	89	10	2016
7	Muhammad Ilmawan	7	13	1.75	246	13	2020
8	Jan G. Jakobsson	7	10	0.875	119	14	2016
9	Firzan Nainu	7	10	1.75	237	10	2020
10	Joseph F. John, Jr.	6	10	0.75	111	11	2016
11	Yee Wan Lee	6	9	0.6	102	9	2014
12	Aaron Lun	6	6	0.75	1052	6	2016
13	Helnida Anggun Maliga	6	8	2	125	8	2021
14	David Moher	6	9	0.857	180	9	2017
15	Alexander R Pico	6	11	0.6	177	11	2014
16	Ali A. Rabaan	6	7	1.5	208	7	2020
17	Alfonso J. Rodriguez-Morales	6	7	0.75	172	7	2016
18	Sweta Singh	6	7	0.545	90	7	2013
19	Yiwei Wang	6	9	0.6	366	9	2014
20	Ben Busby	5	7	0.625	65	17	2016

Table 8 exhibits the top 20 most impactful authors based on the h-index given by Hirsch (2005). F1000Research journal is one of the premium journals where 5290 authors have published their articles. However, only a few authors have more than a five h-index. “Gary D. Bader” and “Sean Ekins” have the maximum h-index of nine amongst the authors, 12 g-index, 12 publications and 577 and 269 citations, although the first publication came in 2014. “Jürgen Bajorath,” ranked number three in the list, has eight h-index, 19 publications, and has been active in publishing since 2012. Identifying the most prolific authors and their research articles can help future researchers extend their research recommendations, understand their research area, and identify the research gaps, which can help conceptualise the future research problem.

### Bibliographic coupling of authors, countries, and organisations

**Figure 7.**
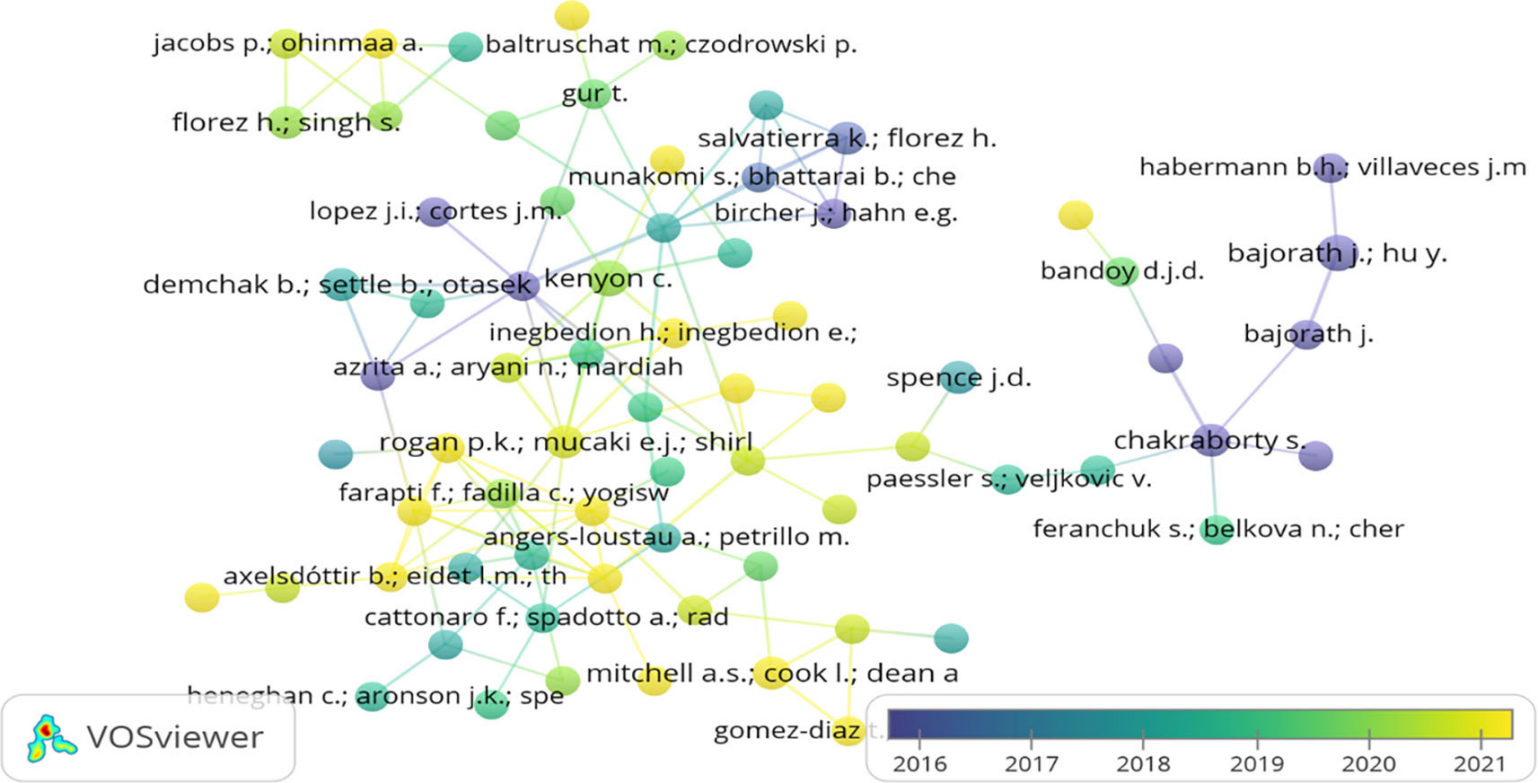
Bibliographic coupling between the authors (Overlay visualisation) (Data source: Scopus, Software: VOS-viewer).

**Figure 8.**
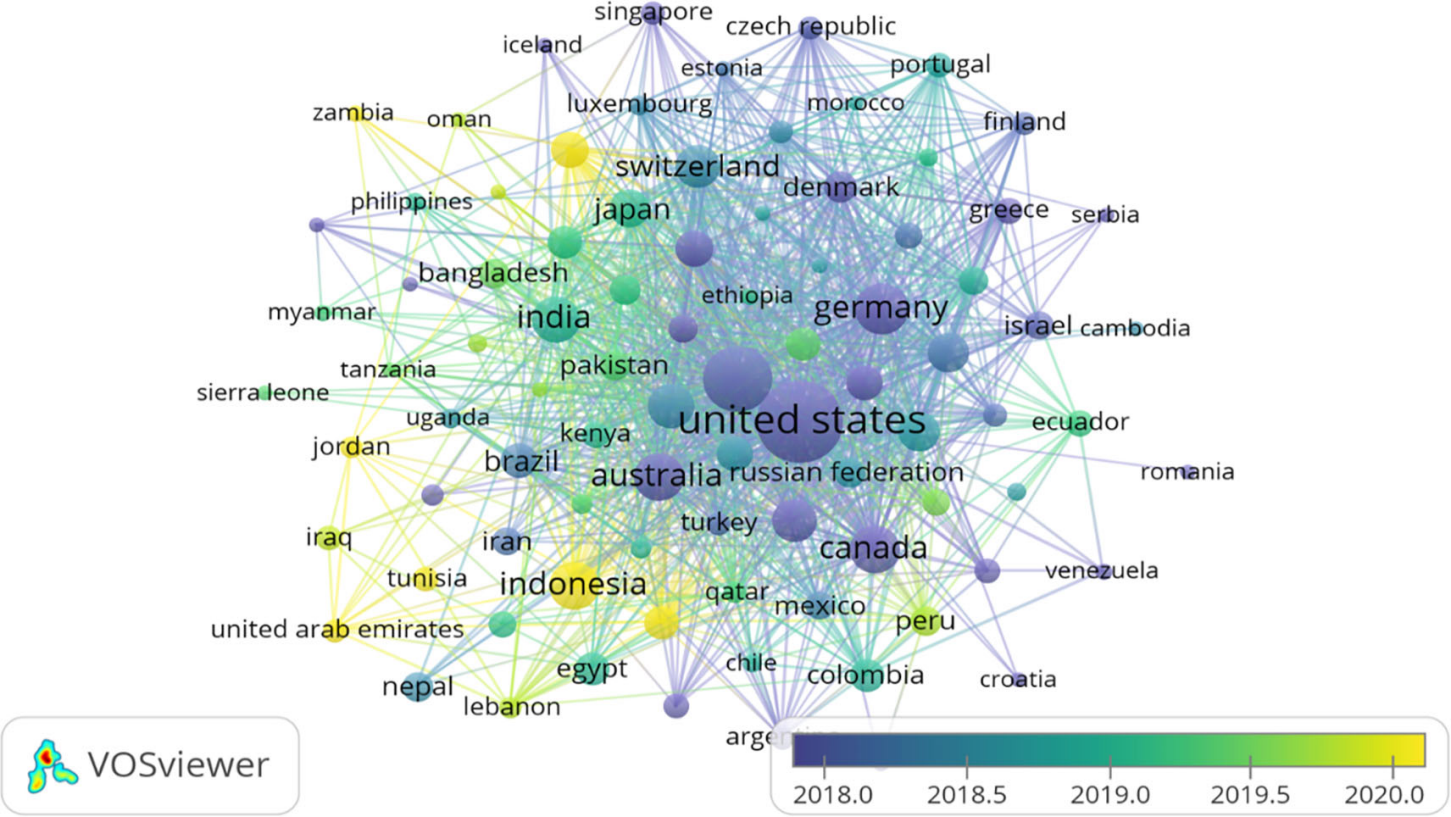
Bibliographic coupling between the countries (Overlay visualisation) (Data source: Scopus, Software: VOS-viewer).

**Figure 9.**
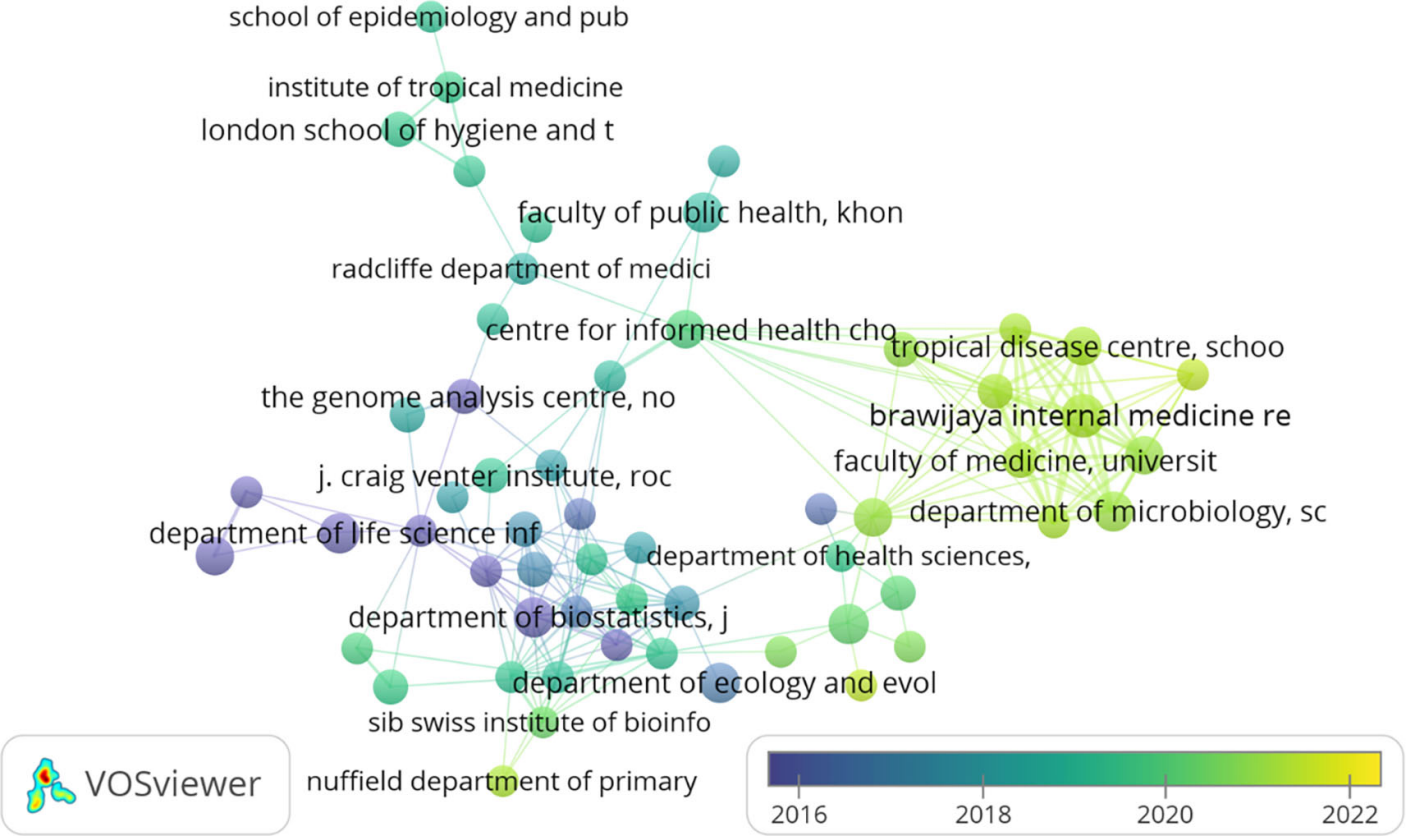
Bibliographic coupling between the organisations (Overlay visualisation) (Data source: Scopus, Software: VOS-viewer).

Bibliographic coupling occurs when two papers cite a third common paper. The two papers address a common subject matter ( Martyn, 1964). In the present study, the bibliographic links between authors, countries and organisations through overlay visualisation are shown in Figure 7, Figure 8, and Figure 9. While examining the bibliographic coupling of authors, the minimum number of documents of an author was restricted to 100. 5290 authors appeared, but only 225 met the desired threshold. However, the largest set of only 71 items connected items were found. Figure 7 depicts the bibliographic coupling between the authors at the various stages of a research journey. Author’s node marked in yellow highlights the youngest/recent (2021) coupling, whereas the authors in purple depicted the oldest (2016) coupling.

Figure 8 shows the bibliographic coupling (overlay visualisation) of countries. During the VOS-viewer bibliometric analysis, countries that published at least five documents were considered for final analysis. Only 89 countries met the desired threshold values out of 164 countries. However, the largest set of connected items was only 88. The United States was found as the most significant contributor in terms of publications as well as citations. They have been involved in publishing papers marked in purple for a long time. In contrast, Asian countries like Indonesia, United Arab Emirates and Qatar are new in bibliographic coupling (marked in yellow).

Figure 9 shows a bibliographic coupling (overlay visualisation) of the organisation. While performing the analysis using VOS-viewer, an organisation published at least four documents that were considered for the final analysis. Out of 14646 organisations, only 83 met the desired threshold criteria, but only 57 organisations had the largest set of connected items. Organisations marked in yellow are the youngest, and those marked in purple are the oldest in the F1000Research journal.

### Emerging themes and trending topics

**Figure 10.**
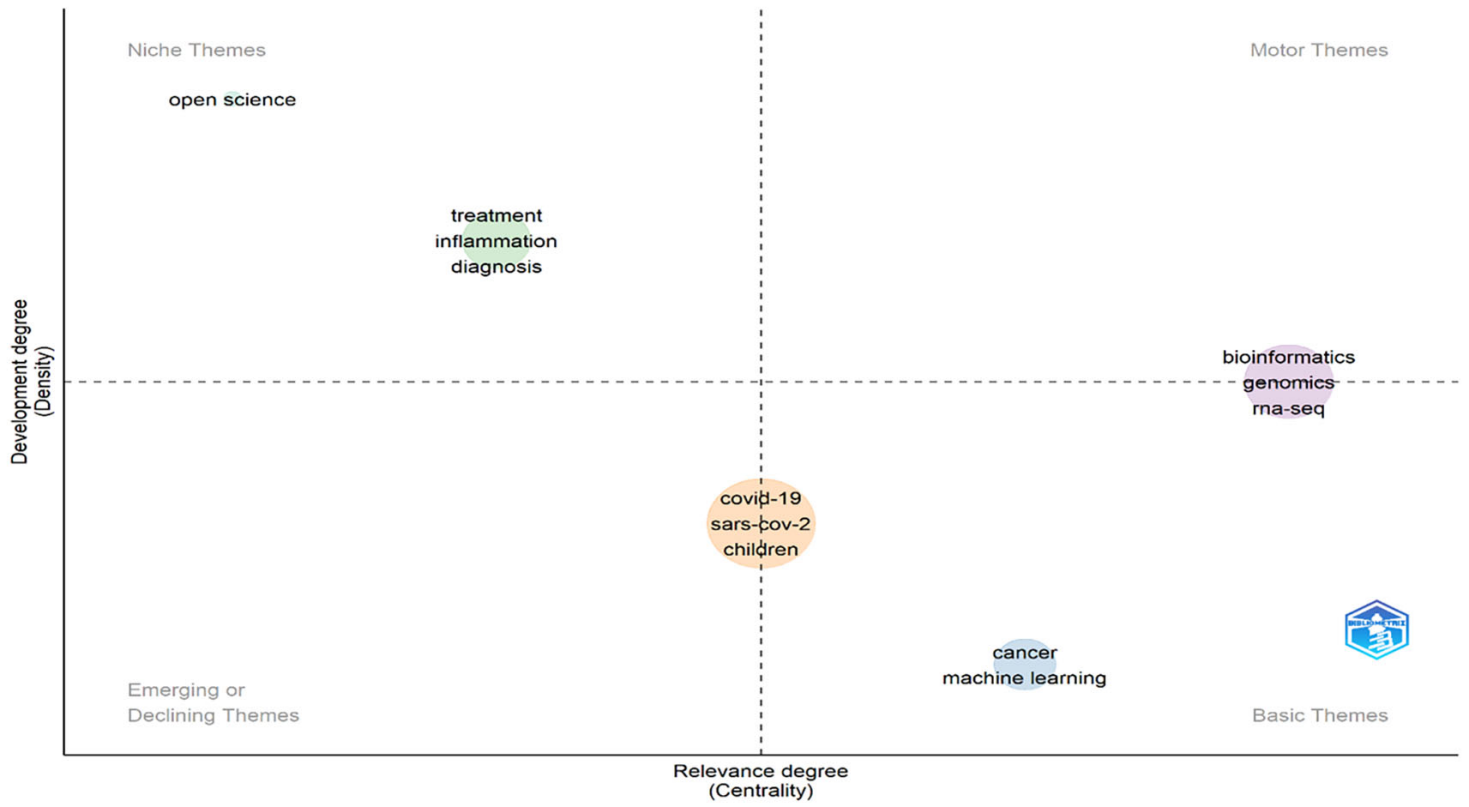
Emerging theme identification (Data source: Scopus, Software: Biblioshiny – R Studio).

Keywords that appeared during the analysis in VOS-viewer software or Biblioshiny (R-studio) were visualised through theme generation. In the present study, Biblioshiny (R-studio) software was used to understand the various themes (niche theme, motor theme, basic theme and emerging or declining theme), which have been divided into four quadrants (Q1, Q2, Q3, and Q4) ( Cobo et al., 2011), identified based on centrality (X-axis) and density (Y-axis). The degree to which a topic is connected to other topics and, in turn, significant in a particular domain is measured by centrality, which assesses the level of inter-cluster relationships. The density, conversely, gauges the degree of intra-cluster cohesion, or more specifically, how closely related the keywords in a given cluster are to one another and how strongly a theme is established ( Forliano et al., 2021). In Figure 10, the upper left (high density and low centrality) includes niche research themes containing the keywords “open science,” “treatment,” “inflammation,” and “diagnosis.” Niche themes suggest it is internally well developed but unable to influence others due to low centrality. Motor themes appeared in the upper right quadrant (high in density and centrality) and included the keywords “bioinformatics,” “genomics,” and “RNA-seq.” It suggests that themes are well-developed and highly influence the researcher. Basic themes appeared in the lower right quadrant (high in centrality and low in density), showing the themes are extending or lying across for discipline and can influence the other researcher/topics but are underdeveloped. Emerging or declining themes appeared in the bottom left quadrant (low centrality and density), which is neither well developed nor influenced by the researcher. Keywords that appeared here are “COVID-19,” “SARS-CoV-2,” and “Children” are a matter of great concern in the present scenario. Based on the thematic map analysis, it can be concluded that the keywords that appeared in niche themes are well-developed and highly influenced by the researchers. In contrast, future researchers need more focus on emerging or declining themes to develop a concrete plan to fight against these widespread (COVID-19, SARS-CoV-2, and children) keywords.

**Figure 11.**
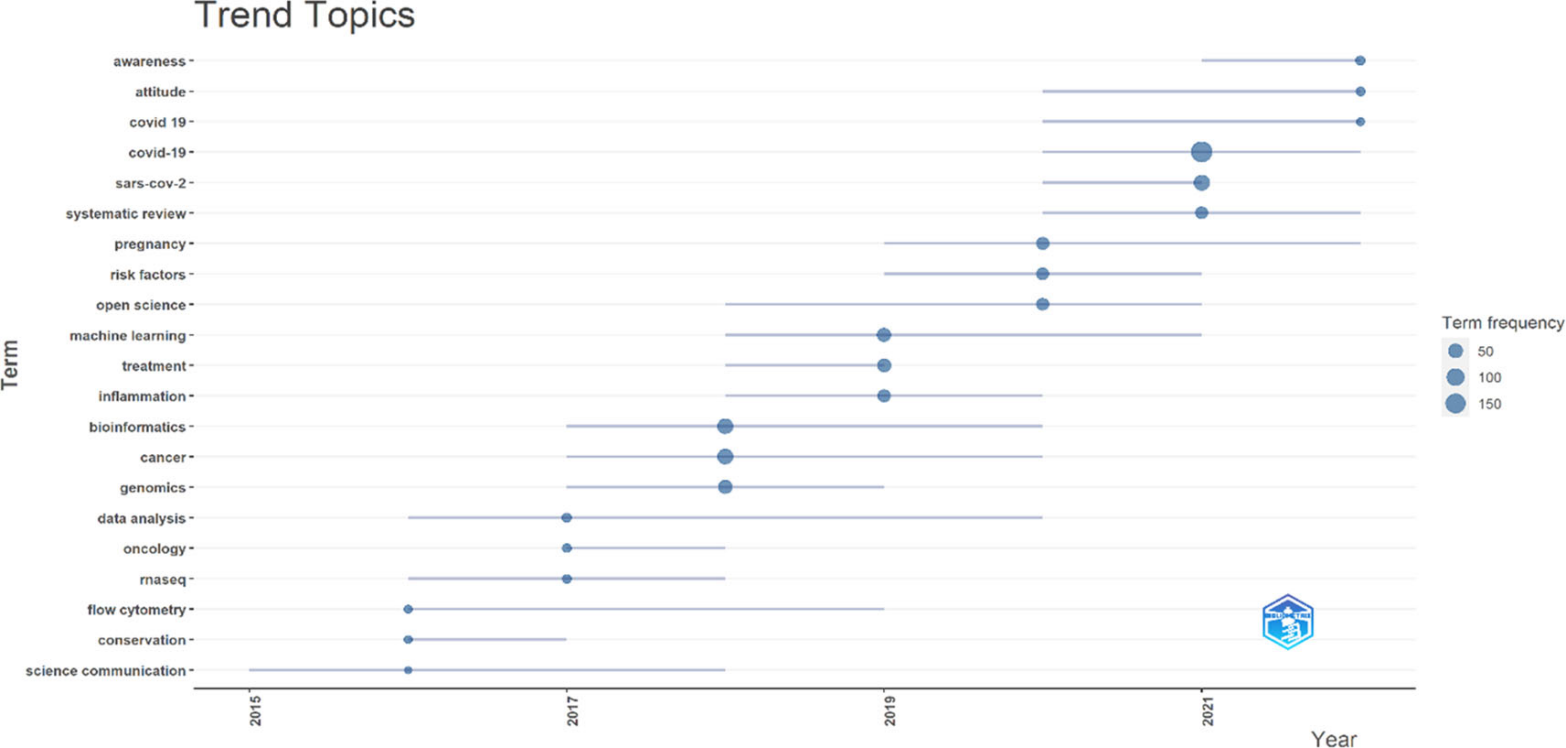
Trending topics (Data source: Scopus, Software: Biblioshiny – R Studio).

The present study visualises trending topics through Biblioshiny (R-studio), shown in Figure 11. Bubbles of the smallest size have shown a minimum of 50, middle 100, and biggest 150 appearances. Recently appeared topics (between 2021-22) are awareness, attitude and COVID-19, although their bubble size is small (minimum <50 and >100 appearances). COVID-19 again appeared between 2020-21 with the biggest bubble (<150 appearances) along with “SARS-Cov-2” (<100 appearances) and “systematic review” keywords. These topics emerged based on keywords in the author’s articles published in the F1000Research journal journey since its inception.

## Discussion: Direction for future research

Bibliometric analysis suggests that future researchers/scholars for developing advanced theories and scholarly practices and utilising them for policy-making decisions ( Mukherjee et al., 2022). Developing advanced approaches directly relates to the keywords' co-occurrences, which appeared during the bibliometric analysis. The present study generated seven clusters based on the keyword’s relevance and its TLS. In all seven clusters, majorly appeared keywords are “Bioinformatics,” “Treatment,” “Children,” “COVID-19,” “Cancer,” “Inflammation,” and “Systematic review”, on which researchers have worked recently. These areas can be further used to discuss the impact and applicability of improving health and immunity to fight against various life-threatening diseases.

*Bioinformatics (Cluster 1 – Red colour):* As per the Oxford English Dictionary, “Bioinformatics” is a conceptualisation of biology in molecular terms (physical chemistry), or it is a molecular information system of molecular biology. In medical science, it has various aims, such as – allowing researchers to access the current information and submission new information, developing tools which can be helpful in data analysis, and utilisation of the developed tools for data interpretation in a meaningful (biological) manner ( Luscombe et al., 2001). The machine learning algorithm can be used to understand the term “Bioinformatics”, which can be helpful in education. Future studies can be done on software designing and data sharing areas through visualisation and “R” to explore the new facts of “Bioinformatics”.

*Treatment (Cluster 2 = Green colour):* A healthy human being finds a physical change in the body, initially undetectable and later become detectable by laboratory testing through clinical trials by physicians during diagnosis ( Scheuermann et al., 2009). There are various diseases caused due to infection, such as tuberculosis which can be deadly and epidemic if not diagnosed at the initial stage ( Ahmad & Mokaddas, 2010). Vaccination is one of the milestone achievements to solve the problem permanently instead of going for treatment and remedy of diseases ( Quilici et al., 2015). Future researchers should focus on proper diagnosis, vaccine, and pathogen identification for curing diseases and developing antibiotics through neuroimaging.

*Children (cluster 3 = Blue colour):* Worldwide, childhood obesity has increased significantly during the past few decades ( Han et al., 2010). Paediatricians should be worried about childhood obesity and act fast to adopt therapies because it accounts for most adult obesity ( Daniels et al., 2015). Children who are obese can develop type 2 diabetes, insulin resistance, and psychosocial problems. Additionally, it has been associated with greater adult morbidity and mortality. Obesity prevention is essential and can be effectively treated by food planning and increasing physical activity ( Lifshitz, 2008). People in urban areas are more prone to asthma and obesity ( Johnson et al., 2010). Asthma and hypertension are also prevalent diseases nowadays; both can be seen together. Prior research has suggested that asthmatic patients are more prone to hypertension than non-asthmatic patients ( Lee et al., 2009). Further research is needed to understand the relationship between children, asthma, obesity, hypertension, stroke, and environmental risk.

*COVID-19 (cluster 4 = Yellow colour):* The COVID-19 pandemic brought the world to its knees because of its uniqueness and communicability. It has taken an important place in our daily routine. It became the most significant concern for the nations and society, and countries like India and China ranked no.1 and 2 in population, were assumed as the most affected countries by SARS-CoV-2. COVID-19 has highly impacted the heart surgeon clinical practice, suggesting the medical team's preparedness to tackle the challenges exposed by SARS-CoV-2 ( Pericàs et al., 2020). Since the outbreak is not over, future researchers should evaluate and monitor everything carefully for a better understanding of the epidemiological properties of COVID-19, which can help develop mechanisms to safeguard public health.

*Cancer (cluster 5 = Purple colour).* Cancer is one of the fastest-growing diseases worldwide ( Popat et al., 2013) in various types, such as breast, blood, liver, lung, ovarian, prostate, etc. Underarm deodorant, specific chemical’s regular and long-term use was found as one of the factors behind the development of breast cancer (under investigation) ( Darbre, 2009). Gene mutation plays a crucial role in predisposition to breast cancer. However, understanding the genes pathways can play a vital role in developing a preventive target to fight against breast cancer ( Sheikh et al., 2015). Few studies suggested immunotherapeutic strategies can help fight against the deadly breast cancer disease ( Marra et al., 2019). Various clinical trials are developing strategies to counter this growing concern; therefore, future researchers can focus on gene mutation, immunotherapy, and reviewing cancer case reports to understand this deadly disease.

*Inflammation (cluster 6 = Cyan colour).* Inflammation studies can play a crucial role in understanding the origin and progression of the disease. A better understanding of the trigger mechanisms that cause inflammation is required to keep enough control over the inflammatory cascade ( Schmid-Schönbein, 2006). Mitochondria are found as the master regulators and controllers of inflammation, but further research is needed to understand the mitochondrial functions as a controlling organelle in inflammatory reactions in patients ( Marchi et al., 2023). Future researchers can focus on bioengineering analysis which can help open the door for inflammation treatment through new and improved interventions ( Schmid-Schönbein, 2006).

*Systematic Review (cluster 7 = Orange colour).* Most of the studies in the top-cited journals are either performed through systematic review or meta-analysis approach; therefore, it is recommended that future research should focus more on clinical trials or quantitative methods.

## Conclusion

The present study attempts to present the publication journey of the F1000Research journal from its inception (2012) to 31 ^st^ December 2022 through bibliometric analysis using VOS-viewer and Biblioshiny (R studio) interface. The publication trends and journal citation analysis were understood and exhibited in Figure 1. The journal gained popularity in 2015 and peaked in 2018 (published 915 articles).

The leading authors, leading organisations, highly cited documents, and leading countries’ contributions are also presented. Future researchers can collaborate with leading authors, organisations, and countries to escalate the work and extend their future recommendations in an unexplored area. Author keywords are presented through web map analysis which can be helpful for the future researcher to explore the unknown or least explored areas in their study and can be correlated with the theoretical concept. The prospective researcher can use the least explored keywords to understand the area minutely and its relationship with future keywords and their impact.

This study has also explored the significant clusters (seven) based on keywords co-occurrence analysis. It has been found that “COVID-19” has the maximum occurrences and highest TLS. “COVID-19” is a significant area of concern for the entire world. Therefore, researchers worldwide focus more on its impact on human beings, treatment, and vaccine development. Bioinformatics is one of the areas which is gaining popularity in the present context. Hence, future researchers can contribute their work to understanding medical science through machine learning and software.

The present study would help future researchers to understand the emerging medical science domain. It will also help the editors and journal to focus more on developing or emerging areas and to understand their importance towards society. Countries which have not contributed even a single article to the F1000Research journals are also a matter of concern; therefore, the editor and publisher must target those countries by providing some financial discount as the journal is on an open-access platform. Future research needs to be planned in virus immunology, virology, and bioinformatics through a clinical trial can be a milestone in medical science. Future researchers can contribute their quality research studies, focusing on emerging themes. It has been observed that there are very few articles having an h-index of more than 5. These authors’ research can guide future researchers to develop their research area around the most impacted articles. They can collaborate with them to bring that emerging theme a way forward.

The present study has also encountered some limitations during the data extraction and analysis stage. VOS-viewer software uses a fractional counting method, whereas the Scopus database generates metadata under a full counting system.

Biblioshiny (R-studio) interface showed poor keywords details, a completely missing number of cited references, and a completely missing Science Categories. Co-occurrence analysis between all keywords and index keywords could not be performed due to poor keywords details.

## Data Availability

Figshare: Metadata extracted from the Scopus database, which is used to study the most impactful countries, authors, organisations, keywords in F1000Research. https://doi.org/10.6084/m9.figshare.22713604 ( Kumar, 2023). Data are available under the terms of the Creative Commons Attribution 4.0 International license (CC-BY 4.0).
