# Supplementary material for: The journey of F1000Research since inception: through bibliometric analysis
Source: F1000Res. 2023 Jun 7;12:516. Originally published 2023 May 18. [Version 2] doi: 10.12688/f1000research.134244.2 (PMC10238821; doi:10.12688/f1000research.134244.2)
Supplement: Peer review history

# Peer review: Reviewer response for version 2

**DOI:** 10.5256/f1000research.150605.r177213

**Published:** 2023-06-12

**Authors:** Ajit Singh

**Affiliations:** 1 Chandigarh University, Sahibzada Ajit Singh Nagar, Punjab, India

No further comments to make.

Is the work clearly and accurately presented and does it cite the current literature?

Yes

If applicable, is the statistical analysis and its interpretation appropriate?

Yes

Are all the source data underlying the results available to ensure full reproducibility?

Yes

Is the study design appropriate and is the work technically sound?

Yes

Are the conclusions drawn adequately supported by the results?

Yes

Are sufficient details of methods and analysis provided to allow replication by others?

Yes

Reviewer Expertise:

Sustainability, Augmented Reality, Tourism and Hospitality, Technology.

I confirm that I have read this submission and believe that I have an appropriate level of expertise to confirm that it is of an acceptable scientific standard.

# Peer review: Reviewer response for version 1

**DOI:** 10.5256/f1000research.147283.r174375

**Published:** 2023-06-02

**Authors:** Ajit Singh

**Affiliations:** 1 Chandigarh University, Sahibzada Ajit Singh Nagar, Punjab, India

The research paper provides a comprehensive and well-structured analysis of the topic. The abstract successfully captures the study's goals and methodologies. However, the key findings may be added in the section. This addition would offer readers a glimpse into the primary insights, thereby piquing their interest.

 The methodology employed in the study is rigorous and appropriate for the research objectives. However, the analysis could be enhanced by incorporating a thematic evaluation of the keywords. This could be done through Biblioshiny.

 The findings of the research paper are supported by sound evidence and data, enhancing the credibility of the study.

 The paper adheres to ethical considerations and provides appropriate acknowledgments and references.

 I have suggested a few papers that explain the thematic evolution of topics or keywords ^1^^-^^3^. You can consider using them as references.

Is the work clearly and accurately presented and does it cite the current literature?

Yes

If applicable, is the statistical analysis and its interpretation appropriate?

Yes

Are all the source data underlying the results available to ensure full reproducibility?

Yes

Is the study design appropriate and is the work technically sound?

Yes

Are the conclusions drawn adequately supported by the results?

Yes

Are sufficient details of methods and analysis provided to allow replication by others?

Yes

Reviewer Expertise:

Sustainability, Augmented Reality, Tourism and Hospitality, Technology.

I confirm that I have read this submission and believe that I have an appropriate level of expertise to confirm that it is of an acceptable scientific standard.

## References

[1] : The thematic evolution of customer engagement research: a comparative systematic review and bibliometric analysis. *International Journal of Contemporary Hospitality Management* .2021;33(10) : 10.1108/IJCHM-04-2021-0470 3585-3609 10.1108/IJCHM-04-2021-0470

[2] : Two decades of customer experience research in hospitality and tourism: A bibliometric analysis and thematic content analysis. *International Journal of Hospitality Management* .2022;100: 10.1016/j.ijhm.2021.103082 10.1016/j.ijhm.2021.103082

[3] : 30th Anniversary of Applied Intelligence: A combination of bibliometrics and thematic analysis using SciMAT. *Applied Intelligence* .2021;51(9) : 10.1007/s10489-021-02584-z 6547-6568 10.1007/s10489-021-02584-z

# Peer review: Reviewer response for version 1

**DOI:** 10.5256/f1000research.147283.r174372

**Published:** 2023-06-01

**Authors:** Rahul Pratap Singh Kaurav

**Affiliations:** 1 Fortune Institute of International Business, New Delhi, Delhi, India

Overall, the article "The journey of F1000Research since inception: through bibliometric analysis" provides a valuable analysis of F1000Research as a journal and its contributions to the field. Here is some feedback to further enhance the article:

1. Introduction: Consider providing a brief background on the significance of bibliometric analysis and its relevance in understanding the growth and impact of academic journals. This will help readers better understand the context and importance of the study.
2. Abstract: The abstract effectively summarizes the objectives and methods of the study. However, it could be improved by briefly mentioning the key findings of the analysis. This will provide a glimpse of the main insights to attract readers' interest.
3. Methods: Provide more details about the specific bibliometric analysis techniques and tools used, such as VOS-viewer and Biblioshiny. This will help readers understand the methodology employed and replicate the study if desired.
4. Results: While the article mentions various analyses conducted, it would be helpful to provide a concise summary of the key findings under each analysis category. This will make it easier for readers to grasp the main trends and insights without delving into the detailed analysis.
5. Conclusion: The conclusion effectively highlights the potential implications of the study for future researchers, editors, and the journal itself. However, consider expanding on the practical implications and specific recommendations for researchers and editors based on the findings. This will provide actionable insights for readers.
6. References: Ensure that the references are properly cited and formatted according to the appropriate style guide (e.g., APA). This will enhance the credibility and professionalism of the article.

Overall, the article provides a comprehensive analysis of F1000 Research and its journey since its inception. By incorporating the suggested improvements, the article will become more engaging, informative, and actionable for readers interested in bibliometric analysis.

 I have suggested a few references to incorporate in the article. You may use them.

- This one paper will help in explaining the multidisciplinary of the journal: Kaurav & Gupta (2022) ^1^
- These papers will help you verify and validate your paper's methodology section: Kaushal *et al.,* (2021) ^2^, Baber *et al.,* (2023) ^3^, Kanta *et al.,* (2021) ^4^

Is the work clearly and accurately presented and does it cite the current literature?

Yes

If applicable, is the statistical analysis and its interpretation appropriate?

Yes

Are all the source data underlying the results available to ensure full reproducibility?

Yes

Is the study design appropriate and is the work technically sound?

Partly

Are the conclusions drawn adequately supported by the results?

Yes

Are sufficient details of methods and analysis provided to allow replication by others?

Yes

Reviewer Expertise:

Destination marketing, bibliometric analysis, and qualitative research.

I confirm that I have read this submission and believe that I have an appropriate level of expertise to confirm that it is of an acceptable scientific standard.

## References

[1] : Trends in Multidiscipline Management Research: Past, Present and Future of FIIB Business Review. *FIIB Business Review* .2022;11(4) : 10.1177/23197145221136966 382-404 10.1177/23197145221136966

[2] : Artificial intelligence and HRM: identifying future research Agenda using systematic literature review and bibliometric analysis. *Management Review Quarterly* .2023;73(2) : 10.1007/s11301-021-00249-2 455-493 10.1007/s11301-021-00249-2

[3] : Three Decades of Consumer Ethnocentrism Research: A Bibliometric Analysis. *Business Perspectives and Research* .2023;11(1) : 10.1177/22785337221098472 137-158 10.1177/22785337221098472

[4] : WILDLIFE TOURISM: A SYNTHESIS OF PAST, PRESENT, AND FUTURE RESEARCH AGENDA. *ENLIGHTENING TOURISM. A PATHMAKING JOURNAL* .2021;11(2) : 10.33776/et.v11i2.5117 10.33776/et.v11i2.5117
